# Complex management of Fabry cardiomyopathy: a case report on the use of alcohol septal ablation and chaperone therapy

**DOI:** 10.1093/ehjcr/ytae317

**Published:** 2024-07-08

**Authors:** Gabriela Neculae, Lucian Predescu, Oana Popa, Elena Rusu, Ruxandra Jurcut

**Affiliations:** Carol Davila University of Medicine and Pharmacy, Department 4 - Cardio-thoracic pathology, 37 Dionisie Lupu Street, 020021 Bucharest, Romania; Expert Center for Rare Genetic Cardiovascular Diseases, Department of Cardiology, Emergency Institute for Cardiovascular Disease ‘Prof Dr C.C.Iliescu’, 258 Fundeni Street, 022328 Bucharest, Romania; Carol Davila University of Medicine and Pharmacy, Department 4 - Cardio-thoracic pathology, 37 Dionisie Lupu Street, 020021 Bucharest, Romania; Expert Center for Rare Genetic Cardiovascular Diseases, Department of Cardiology, Emergency Institute for Cardiovascular Disease ‘Prof Dr C.C.Iliescu’, 258 Fundeni Street, 022328 Bucharest, Romania; Department of Cardiology, ‘Agrippa Ionescu’ Emergency Hospital, 7 Arhitect Ion Mincu Street, 011356 Bucharest, Romania; Department of Nephrology, Fundeni Clinical Institute, 258 Fundeni Street, 022328 Bucharest, Romania; Carol Davila University of Medicine and Pharmacy, Department 4 - Cardio-thoracic pathology, 37 Dionisie Lupu Street, 020021 Bucharest, Romania; Expert Center for Rare Genetic Cardiovascular Diseases, Department of Cardiology, Emergency Institute for Cardiovascular Disease ‘Prof Dr C.C.Iliescu’, 258 Fundeni Street, 022328 Bucharest, Romania

**Keywords:** Fabry cardiomyopathy, Alcohol septal ablation, Migalastat, Multimodality imaging, Case report

## Abstract

**Background:**

Cardiac involvement in Fabry disease (FD) usually manifests as a concentric left ventricular hypertrophy with rare cases developing left ventricular outflow tract obstruction (LVOTO), symptoms varying from fatigue and exercise associated dyspnoea to angina or arrhythmias.

**Case summary:**

We present the case of a 54-year-old man with cardiovascular risk factors and aggravated exertional dyspnoea in the past year, in whom the echocardiography showed hypertrophic obstructive cardiomyopathy (HOCM). Cardiac magnetic resonance was used as differential diagnosis tool between sarcomeric HOCM and other phenocopies, suggesting a cardiac involvement of FD. Final diagnosis was formulated based on genetic testing and enzymatic activity of α-galactosidase. Left ventricular outflow tract obstruction was addressed by alcohol septal ablation successfully both in short term as well as at 1-year follow-up.

**Discussion:**

The present case illustrates the complex clinical pathway of a patient with HOCM due to FD, where multimodality imaging was instrumental from differential diagnosis to therapeutic choices, which addressed both the pathogenic background and the organ involvement. Although at the moment the number of patients with of FD cardiomyopathy undergoing LVOTO reduction therapies is scarce, current recommendations should be extended to also include these patients.

**Learning points**

- Fabry cardiomyopathy often presents as concentric non-obstructive left ventricular hypertrophy, although there have also been rare case reports of patients with obstructive forms.
- Multimodality imaging is instrumental in the differential diagnosis with other phenocopies of hypertrophic cardiomyopathy (HCM), as well as for establishing good therapeutic choices.
- For Fabry disease-associated hypertrophic obstructive cardiomyopathy, when pharmacological therapy fails to reduce left ventricular outflow tract obstruction or to alleviate symptoms, the recommendations for septal reduction therapies may be similar to those in sarcomeric HCM, including alcohol septal ablation or myectomy.

## Introduction

Fabry disease (FD) is an X-linked inherited disorder of glycosphingolipid metabolism due to deficient or absent lysosomal α-galactosidase A activity.^1^ Classically affected hemizygous males may display all the characteristic central and peripheral neurological, skin,^2^ renal,^3^ cardiovascular, and cochleo-vestibular signs of the disease, while heterozygous females have symptoms ranging from very mild to severe. Non-classical forms can appear in patients with residual enzymatic activity and can be associated with limited organ involvement. Cardiac involvement usually consists of left ventricular hypertrophy (LVH),^4^ with symptoms including palpitations, angina, and dyspnoea.^2^ Although not typical, some patients with FD may develop left ventricular outflow tract obstruction (LVOTO).^4^

## Summary figure

**Figure:**
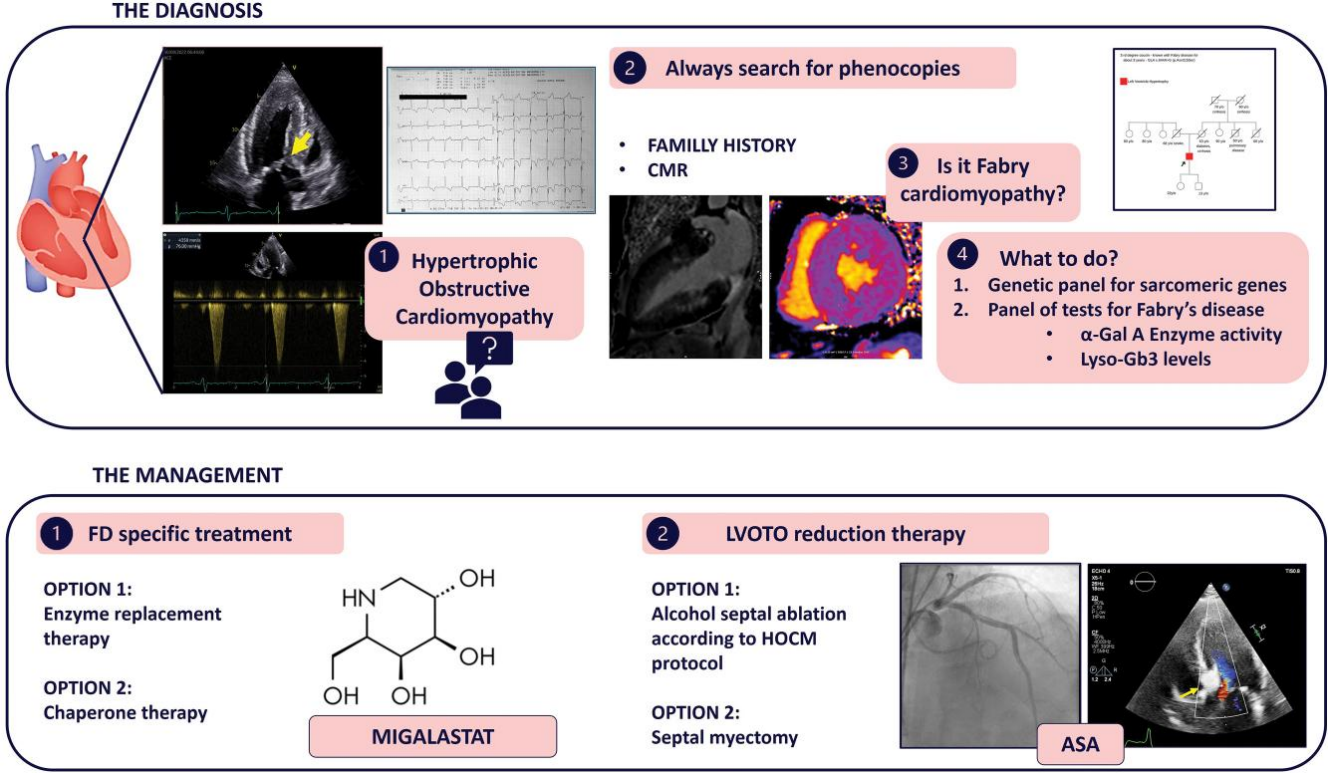


## Case presentation

We present the case of a 54-year-old male with hypertension, dyslipidaemia, presenting with aggravated exertional dyspnoea since August 2021.

On our first examination, the patient describes dyspnoea and chest pain, affecting day-to-day activities, with progressive worsening during the previous year. The genealogy revealed a history of FD and sudden death in third-degree cousins. A complete physical exam showed no angiokeratoma. The patient had never experienced anhidrosis, but he describes occasional acroparaesthesia that is not aggravated by fever, mild tinnitus, and vertigo. He also presented a systolic murmur on the left parasternal border that increased in intensity during Valsalva manoeuvre.

The laboratory workup revealed a slight increase in serum creatinine and microalbuminuria, the estimated glomerular filtration rate (eGFR) being 54 mL/min/1.73 m^2^ (normal range 85–116 mL/min/1.73 m^2^). We also noted elevated levels of N-terminal prohormone of brain natriuretic peptide (6102 pg/mL; normal < 300 pg/m) and high sensitivity troponin I (76.4 ng/L; normal < 29 ng/L).

The electrocardiogram showed sinus rhythm with LVH, left axis deviation, and secondary repolarization abnormalities (Supplementary material online).

**Figure 1:**
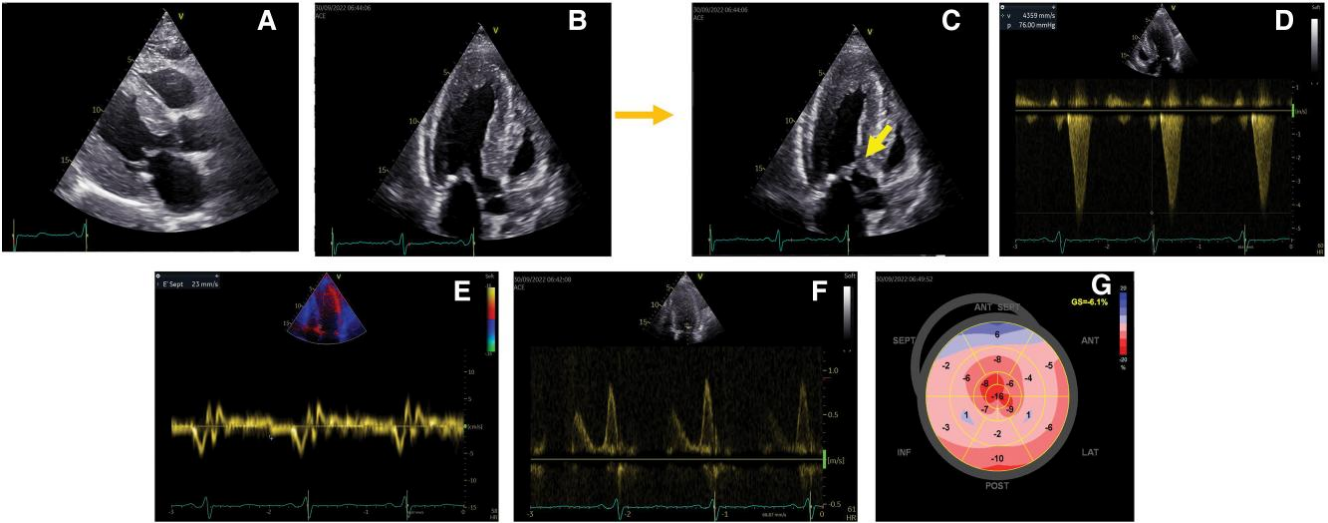
Echocardiographic aspects. (*A*) Parasternal long-axis view showing concentric left ventricular hypertrophy; (*B* and *C*) apical three chambers view showing systolic anterior movement of the mitral leaflet; arrow pointing at contact point with basal septum; (*D*) left ventricular outflow tract gradient 76 mmHg, without provocation manoeuvres; (*E*) low mitral annulus tissue velocities; (*F*) mitral valve left ventricular filing pattern showing *E* < *A*; (*G*) Bull’s-eye plot of global longitudinal strain showing severe longitudinal dysfunction.

**Figure 2:**
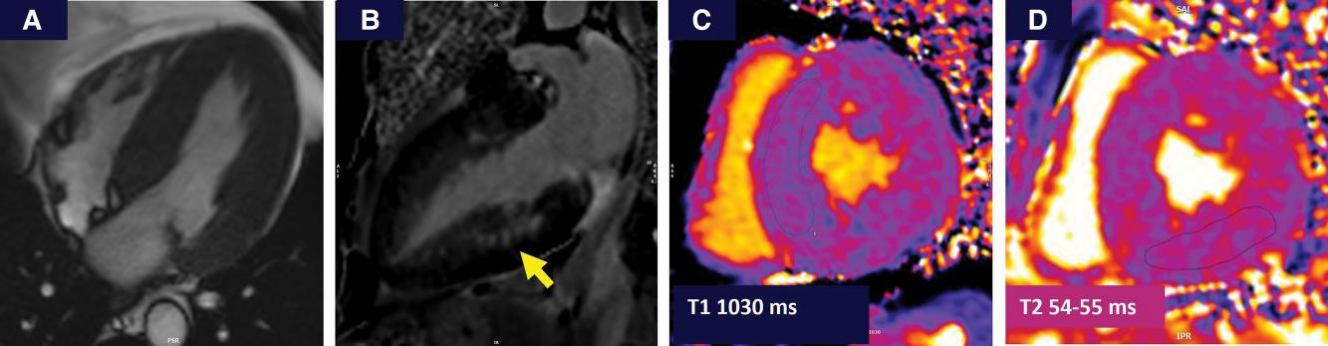
Cardiac magnetic resonance aspects. (*A*) Apical four-chamber view showing concentric severe left ventricular hypertrophy with a maximum wall thickness of 22 mm; (*B*) mid-myocardial late gadolinium enhancement in the basal and mid segments of inferior walls; (*C*) normal native T1 values (1030 ms); (*D*) normal global T2 values (46 ms) and elevated values in late gadolinium enhancement areas (54–55 ms).

Transthoracic echocardiography (*Figure 1*, Supplementary material online) showed severe LVH with a maximum wall thickness of 23 mm at the septal level and systolic anterior movement (SAM) of the mitral valve. A spontaneous gradient of 76 mmHg was identified. We also noted severely impaired LV longitudinal function, with a global longitudinal strain of −6.1% but preserved ejection fraction, as well as impaired diastolic function and left atrial dilation. The right ventricle was also hypertrophied with a free wall thickness of 8 mm. There were no other significant valvular abnormalities and no aortic root dilation. A diagnosis of hypertrophic obstructive cardiomyopathy (HOCM) was maintained and further investigated through cardiac magnetic resonance (CMR) (*Figure 2*), which confirmed severe LVH with mid-wall late gadolinium enhancement in the basal and mid segments of the posterior and inferior wall, associated with a normal T1 and elevated T2 values.

Genetic testing using next-generation sequencing with a cardiomyopathy-dedicated panel was performed, and a pathogenic mutation in the GLA gene (p.Asn215Ser) was detected, with no concomitant sarcomeric variants. At further testing, our patient had a small residual lysosomal enzyme activity and an elevated lyso-GL-3. Based on these data, the final diagnosis was FD with predominantly cardiac involvement.

**Figure 3:**
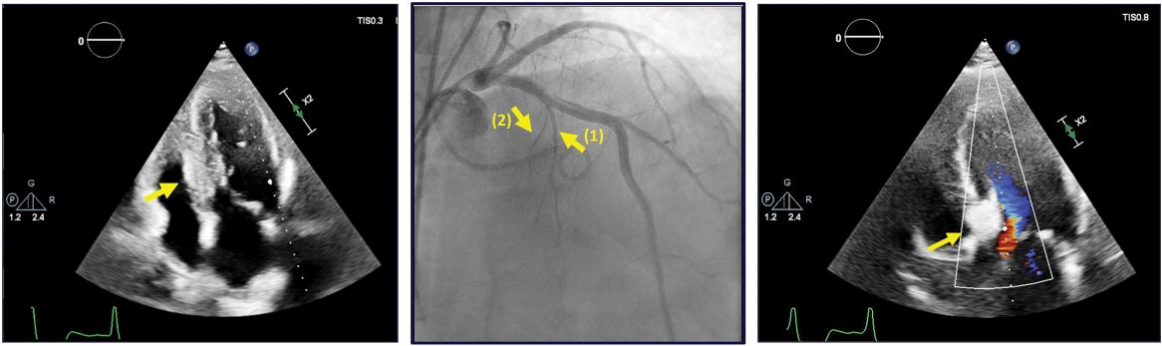
Intra-procedural aspects. Middle panel showing angiographic aspects of the septal branches, with early bifurcation. First panel shows contrast disposition (arrow) in the whole septum including towards the right ventricle after contrast was administered in the septal branch marked with (1). Third panel shows contrast disposition (arrow) limited to the basal septum after contrast was administered in the septal branch marked with (2).

**Figure 4:**
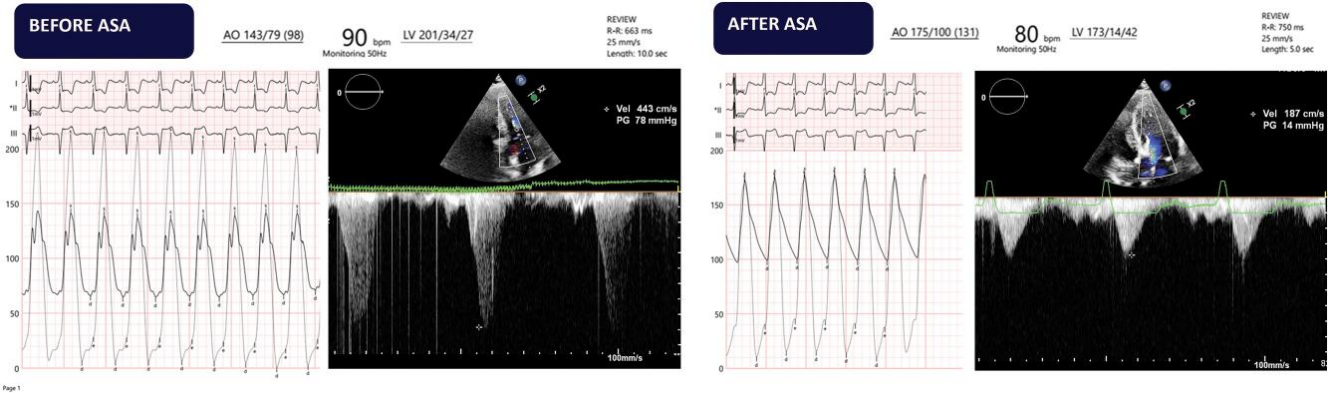
Intra-procedural aspects: pressure curves measured trough hear catheterization and left ventricular outflow tract gradient measured trough Doppler echocardiography before and after alcohol septal ablation, showing significant reduction in gradient.

In terms of management, based on the amenability of the genetic variant, our patient was started on specific FD chaperone therapy with migalastat, as well as symptomatic therapy of HOCM with high-dose beta-blockers. As even under maximal treatment, he remained very symptomatic, LV septal reduction therapy was taken into consideration. Based on the patient’s preference and mitral valve anatomy, we planned to perform an alcohol septal ablation (ASA). Using contrast-enhanced echocardiography (*Figure 3*) to identify the septal artery that supplies the basal septum, we established the most well-suited septal branch and proceeded to inject 2 mL of alcohol. At the end of the procedure, a significant response was noted, with a reduction of the LV outflow tract (LVOT) gradient from 76 to 14 mmHg. The post-procedural evolution was favourable, with no complications. After the procedure, we noted an improved LVOT haemodynamics, with less SAM and a maximum LVOT gradient of 23 mmHg at 48-h follow-up (*Figure 4*). Beta-blocker treatment was restarted. At 1-year follow-up, the patient is asymptomatic, with normal exercise capacity and maintaining a maximal LVOT gradient < 25 mmHg at both rest and provocation manoeuvres (videos provided in Supplementary material online). Also, we observed an improvement of eGFR to 69 mL/min/1.73 m^2^ and normalization of albuminuria. Lysosomal enzyme activity increased slightly, and lyso-GL-3 decreased.

Given the fact that FD is an X-linked disease, we provided family screening for our patient’s daughter. The now 20-year-old has the mutation in a heterozygous form with normal enzymatic activity and no phenotypic features.

## Discussion

This is a very rare case of FD associated with HOCM, which was successfully managed by a complex combination of ASA and chaperone therapy (*Summary figure*).

The main feature of cardiac involvement in FD is LVH, with a series of particularities potentially leading the cardiologist towards a FD diagnosis. These features include asymmetric LVH with septal thickening and progressive thinning of the posterior wall,^5^ papillary muscle hypertrophy,^6^ and longitudinal dysfunction in the posterior and inferior segments.^7^ Cardiac magnetic resonance may raise suspicion of FD through tissue characterization. In FD patients, native T1 sees an evolution from normal values in the early LysoGb3 accumulation phase to low T1 during focal inflammation and myocyte hypertrophy phase and then again normalization of T1 when fibrosis occurs.^8^ As in our case, late phases of the disease do not associate low T1 values anymore due to extensive fibrosis; however, elevated T2 provides a marker of associated inflammation and correlates to high troponin values.^9^

The mutation found in our patient is usually correlated with late-onset cardiac phenotype and some residual enzymatic activity.^9^ The GLA variant p.N215S determined in our patient a phenotype with concentric LVH and LVOTO and normalization of native T1 on CMR. Given the atypical phenotype, we ought to mention the pivotal role of family history in the diagnosis process. Although no history of disease was present in first- and second-degree relatives, a thorough interview has provided a critical clue in this case. As beta-blocker treatment failed to reduce outflow tract obstruction or to alleviate symptoms in our patient, the recommendations for treatment of LVOTO were extrapolated from those in sarcomeric HOCM. Current guidelines recommend LVOTO reduction therapy for patients with a gradient above 50 mmHg, and although myectomy rather than ASA is recommended, in experienced centres, the two procedures have similar outcomes based on careful patient selection.^10^ Although there are limited data at this moment, there are recent case series published to showcase the efficacy of ASA in patients with FD and obstructive phenotype, showing an acute haemodynamic effect that persisted during subsequent follow-up at 6 months.^11^ In the Romanian FD cohort of 73 patients, there are seven with p.N215S mutation, three of them presented with LVOTO, and two of them underwent successful surgical myectomy with beneficial long-term results.^4^

Pathogenic therapy was also used as indicated in a male patient with FD,^12^ with a choice among enzyme replacement or chaperone therapy when an amenable variant is present. In our patient, migalastat was chosen as it has favourable outcome data, as well as studies showing a possible regression of the LV mass under treatment.^13^

The present case illustrates the complex clinical pathway of a patient with HOCM due to FD, where multimodality imaging was instrumental from differential diagnosis to therapeutic choices, which addressed both the pathogenic background and the organ involvement.

## Supplementary Material

ytae317_Supplementary_Data

## Data Availability

There are no new data associated with this article.

## Lead author biography

Gabriela Neculae is a cardiology fellow at the Emergency Institute for Cardiovascular Disease Prof. Dr. C. C. Iliescu, with clinical and research training at the Expert Center for Rare Genetic Cardiovascular Diseases under the guidance of Prof. Dr Ruxandra Jurcut. She is interested in the evaluation and management of patients with hereditary heart disease, particularly infiltrative and storage myocardial disease. She is a PhD student under the coordination of Prof. Dr Ruxandra Jurcut, researching effectiveness of modern treatment options in cardiac amyloidosis.
